# Engineered neurogenesis in naïve adult rat cortex by Ngn2-mediated neuronal reprogramming of resident oligodendrocyte progenitor cells

**DOI:** 10.3389/fnins.2023.1237176

**Published:** 2023-08-17

**Authors:** Stanley F. Bazarek, Mentor Thaqi, Patrick King, Amol R. Mehta, Ronil Patel, Clark A. Briggs, Emily Reisenbigler, Jonathon E. Yousey, Elis A. Miller, Grace E. Stutzmann, Robert A. Marr, Daniel A. Peterson

**Affiliations:** ^1^Center for Stem Cell and Regenerative Medicine, The Chicago Medical School, Rosalind Franklin University of Medicine and Science, North Chicago, IL, United States; ^2^Center for Neurodegenerative Disease and Therapeutics, The Chicago Medical School, Rosalind Franklin University of Medicine and Science, North Chicago, IL, United States

**Keywords:** oligodendrocyte precursor cell, NeuroD1 transcription factor, Neurogenin 2, NG2 cell, reprogramming and differentiation, neuronal replacement, neural stem/progenitor cells

## Abstract

Adult tissue stem cells contribute to tissue homeostasis and repair but the long-lived neurons in the human adult cerebral cortex are not replaced, despite evidence for a limited regenerative response. However, the adult cortex contains a population of proliferating oligodendrocyte progenitor cells (OPCs). We examined the capacity of rat cortical OPCs to be re-specified to a neuronal lineage both *in vitro* and *in vivo*. Expressing the developmental transcription factor Neurogenin2 (Ngn2) in OPCs isolated from adult rat cortex resulted in their expression of early neuronal lineage markers and genes while downregulating expression of OPC markers and genes. Ngn2 induced progression through a neuronal lineage to express mature neuronal markers and functional activity as glutamatergic neurons. *In vivo* retroviral gene delivery of Ngn2 to naive adult rat cortex ensured restricted targeting to proliferating OPCs. Ngn2 expression in OPCs resulted in their lineage re-specification and transition through an immature neuronal morphology into mature pyramidal cortical neurons with spiny dendrites, axons, synaptic contacts, and subtype specification matching local cytoarchitecture. Lineage re-specification of rat cortical OPCs occurred without prior injury, demonstrating these glial progenitor cells need not be put into a reactive state to achieve lineage reprogramming. These results show it may be feasible to precisely engineer additional neurons directly in adult cerebral cortex for experimental study or potentially for therapeutic use to modify dysfunctional or damaged circuitry.

## Introduction

Direct *in vivo* reprogramming of resident tissue cells to re-specify cell lineage has been achieved in the pancreas (Zhou et al., 2008) and heart (Qian et al., 2012; Song et al., 2012), providing a potential approach to therapeutic cell replacement that could avoid the immunogenicity, time, and expense of grafting exogenous cells (Bazarek and Peterson, 2014; Torper and Gotz, 2017). However, neurons are specified into highly distinct subtypes (Molyneaux et al., 2007) and effective neuronal replacement will require engineering authentic, subtype-specific neurons (Bazarek and Peterson, 2014). Whereas neuronal turnover has been demonstrated in adult human hippocampus (Eriksson et al., 1998) and striatum (Ernst et al., 2014), the human cerebral cortex is normally devoid of adult neurogenesis (Kornack and Rakic, 2001; Bhardwaj et al., 2006) with a limited regenerative response reported following ischemic cortical injury (Jin et al., 2006; Lindvall and Kokaia, 2015).

The demonstration that expressing select developmental transcription factors in cultured mouse astrocytes resulted in their conversion to functional, subtype-specific neurons (Heins et al., 2002; Heinrich et al., 2010; Kempf et al., 2021) suggested that direct *in vivo* conversion of resident glia to neurons could be a potential approach for brain repair Indeed, a growing number of studies show *in vivo* conversion of astrocytes to various neuronal subtypes in mouse spinal cord (Tai et al., 2021; Zhou et al., 2021) and mouse striatum (Giehrl-Schwab et al., 2022; Zhang et al., 2022). Reprogramming in the cerebral cortex and hippocampus reportedly requires the targeted glia to be in a reactive state to achieve neuronal induction (Buffo et al., 2005; Grande et al., 2013; Guo et al., 2013; Heinrich et al., 2014; Lentini et al., 2021). However, injury responses are highly variable and poorly defined and may not feature in dysregulated circuitry, such as epilepsy, addictive disorders, and psychiatric disorders, where newly engineered neurons could play a modulatory role (Southwell et al., 2014).

Despite reports of success with neuronal reprogramming in a number of brain regions, approaches to direct *in vivo* reprogramming have recently come under criticism. The controversy largely stems from a lack of certitude that pre-existing neurons were not inadvertently targeted, as this would make interpretation of the outcomes problematic (Calzolari and Berninger, 2021; Wang et al., 2021; Chen et al., 2022; Cooper and Berninger, 2022). Suggested considerations to allay concerns in further studies included demonstrating cell-specific targeting of reprogramming factors and demonstrating progression from early to late neuronal lineage states as confirmation of an induced neuron.

With this perspective in mind, we undertook a study to reprogram oligodendrocyte progenitor cells (OPCs). OPCs, also known as NG2 Glia, represent an attractive alternative to astrocytes as a target cell for neuronal reprogramming. OPCs have a diverse developmental origin that may contribute to their apparent regional heterogeneity in the adult CNS, with cortical OPCs arising postnatally from the dorsal ventricular zone (Newville et al., 2017). OPCs are characterized by their expression of Olig2, Sox10, NG2, and Pdgfa. Definitive OPCs gradually reduce expression of NG2 and Pdgfa upon progressing to terminal differentiation as oligodendrocytes (Nishiyama et al., 2016). OPCs are the largest dividing cell population in the healthy, naive adult cortex (Dawson et al., 2003), respond to injury (Simon et al., 2011; von Streitberg et al., 2021), and homeostatically maintain a latticed distribution (Hughes et al., 2013) throughout the entire cortical region. As their primary role is understood to be a reserve population to proceed to terminal differentiation as oligodendrocytes when needed, OPCs offer an abundant and renewable cell population for neuronal reprogramming.

Here we show that retroviral gene delivery of the transcription factor neurogenin2 (Ngn2) can reprogram isolated adult rat OPCs into functional neurons and produce phenotypically mature neurons from naïve OPCs in the adult rat cortex. We isolated gray matter OPCs from adult rat cortex and screened developmental transcription factors for functional neuronal induction capacity (Heinrich et al., 2011). Ngn2 reprogrammed OPCs into functional neurons *in vitro* and subsequent *in vivo* expression of Ngn2 in normally proliferating naive OPCs reprogrammed these cells through a transitional immature neuronal phenotype into mature glutamatergic pyramidal neurons with authentic dendritic arbor, axonal extension, and synaptic contacts from pre-existing neurons. Our results demonstrate direct *in vivo* engineering of mature neurons in multiple areas of the naive adult rat cortex. Engineering precise neuronal subtypes by reprogramming resident glia in this fashion may prove useful for experimental and potentially therapeutic manipulation of neuronal circuitry in the intact brain and for endogenous cell replacement following disease or neurological injury.

## Materials and methods

### OPC isolation and culture

Adult Hooded Long Evans Rats (*n* = 4) were deeply anesthetized, guillotined, and brains removed. Brains were cut into 2 mm slabs with a coronal brain matrix and the neocortex carefully dissected to avoid inclusion of white matter under a dissecting microscope in chilled HBSS buffer. Tissue was dissociated using a papain-based neural dissociation kit (Miltenyi) and cells were selected for the O4 antigen via Magnetic Activated Cell Sorting (MACS) and cultured as described previously (Dincman et al., 2012). OPCs were grown and passaged on PDL/laminin coated plastic dishes or glass coverslips in OPC growth medium (DMEM/F12, BSA (0.1%), N2 (1%), PDGFaa (10 ng/mL), FGF2 (20 ng/mL), and Insulin (5 μg/mL)) at 5% CO_2_. Cells were passaged, cryopreserved, and maintained as a primary cell line with no later than passage 7 used in experiments.

### Differentiation assays

Differentiation potential was assessed by replacing OPC growth media with Oligodendrocyte Differentiation Media (DMEM/F12, N2 (1%), B-27 Supplement (B27 1%), Penicillin/Streptomycin/Fungizone (PSF 1%), Insulin (50 ng/mL), Triiodothyronine (T3 40 ng/mL)) or Astrocyte Differentiation Media (DMEM/F12, Fetal Bovine Serum (10%), PSF (1%)). Experiments were performed in triplicate.

### Neuronal reprogramming

OPCs were transduced with OPC growth media containing retroviral constructs carrying the transgene for candidate neurogenic transcription factors (Supplementary Table S1). *In vitro* screening studies initially used a mixture of the constitutive CAG promoter or the OPC-specific NG2 promoter in different constructs. No advantage was conferred by NG2-promoter expression and to avoid potential silencing with neuronal differentiation, we chose to standardize on the CAG promoter for our studies. After 24 h, transduction media was replaced with Neuronal Reprogramming Media (Neuralbasal Media, B27 (2%), Glutamax (1%), PSF (1%)) at 10% CO2 with BDNF (20 ng/mL) added on Day 5 and every 4 days thereafter with no media changes (Heinrich et al., 2011). For co-culture experiments, PN1 rat neurons (derivation described in Sun et al., 2008) were plated onto reprogrammed neurons within 48 h of their transduction. Experiments were performed in triplicate. Time-lapse imaging studies were conducted using a Leica SP8 resonance confocal microscope equipped with an environmental stage (Okolab) and cultures were maintained at 37°C and 5% CO_2_ for the duration of the imaging session.

### Immunofluorescence staining for cell culture

Coverslips were transferred and rinsed in a well of PBS and fixed in 4% paraformaldehyde (0.1 M phosphate buffer). Three rinses in Tris-buffered saline (TBS) preceded a 1 h block in 5% donkey serum/0.25% TritonX-100/TBS solution (TBS++). For multiple immunostaining, sections were incubated overnight (at 4°C on a shaker table) with primary antibodies diluted in TBS with 0.25% TritonX-100 (TBS+). Primary antibodies used are listed in Supplementary Table S2. Following two 30 min block/rinses with TBS++, sections were incubated for 2 h with Alexa Fluor 488 (Molecular Probes), Cy3, or Cy5 (Jackson Immunoresearch) diluted in TBS+ (1:500) for 2 h at room temperature. Following two 15-min rinses with TBS, cells were counterstained with DAPI, rinsed with TBS, and coverslips were mounted onto slides, with a glycerol-polyvinyl alcohol plus DABCO (1,4 diazabicyclo [2.2.2] octane; Sigma D2522, Sigma-Aldrich, Inc., St. Louis, MO, United States) PVA-DABCO solution to self-seal and prevent fading, and stored at 4°C in the dark. Negative controls lacking primary antibodies were run along samples.

### *In vitro* quantitation

To assess the efficiency of both viral transduction and neuronal conversion, the number of total cells (DAPI), virally transduced cells (GFP), and converted neurons (beta-III-tubulin) was estimated at each time in the screening assay by systematic sampling of coverslips in triplicate. Coverslips were imaged on an Olympus Spinning Disk (DSU) confocal microscope under the control of stereological software (StereoInvestigator, MBF Bioscience, Inc.) to achieve systematic, fractionated sampling. The entire coverslip was defined as a contour and the sampling density adjusted to acquire images at 10 sites with a randomized start site. This approach assured that all regions of the coverslip had an equal probability of being sampled. Confocal stacks (20 focal planes at 1 μm intervals) were automatically collected by the software at each site for each channel of fluorescence. Cells of each phenotype (DAPI, GFP, and beta-III-tubulin) were counted using the software to obtain an estimate of total cells (DAPI), transduction efficiency (GFP cells over DAPI cells), and conversion efficiency (beta-III-tubulin cells over GFP cells) per coverslip. The extent of colocalization of beta-III-tubulin was also calculated. As the physical parameters of the coverslip did not change, the results are expressed as the sum of the 10 fields sampled. Efficiency calculations were defined as the mathematical ratio of the mean values.

### Molecular analysis

Cells were rinsed with PBS and harvested with no more than 5 × 10^5^ cells resuspended in 1 mL CCM, then lysed and extracted with the RNeasy Plus Micro kit (Qiagen) using larger volumes for the purification of RNA from cells protocol. RNA concentrations were obtained using a Nanodrop 1000 (Thermo Scientific) using 1.5 μL RNA. Each sample was then assessed for quality using the Experion Capillary Electrophoresis System (Bio-Rad). Samples were diluted, denatured and prepared according to the manufacturer’s directions for the Experion RNA HighSens analysis kit (Bio-Rad). They were run on the same chip as an extraction negative control and a positive control and compared using the Bio-Rad Experion software (v3.2.243.0) to a supplied denatured, diluted RNA ladder. Only samples with an RQI (RNA Quality Indicator) number greater than 7.0 were used for arrays. 400 ng of RNA from each sample was reverse transcribed using the ImpromII Reverse Transcription system (Promega, manufacturer’s directions). 91 μL of RNase-free water was added to the cDNA for each sample. The PCR component mix was made up using iTaq Universal SYBR Green supermix (Bio-Rad) for 96-well format A for use in a Bio-Rad iCycler as instructed by the manufacturer (Qiagen). Each sample was pipetted into a Rat Neurogenesis array (Qiagen PARN-404Z) and performed on a Bio-Rad iCycler. The same threshold was set for all arrays and controls checked. The rat genomic DNA contamination control indicated the absence of genomic DNA. The positive PCR control wells were within 2 cycles of a Ct of 20, indicating a lack of inhibitors. Data was analyzed using the RT2 Profiler PCR Array Data Analysis software (v3.5) from Qiagen. Three reference genes that had a difference in Ct values of <2 across all samples were chosen to analyze the data. Delta-delta Ct was calculated for each well to find genes that were under and over expressed by more than 4 fold in the 7dpt and 15dpt day groups when compared to the OPC control cells.

### Electrophysiological recordings

Electrophysiological recordings were conducted at room temperature (22°C) using standard whole-cell patch-clamp techniques, extracellular artificial cerebrospinal fluid gassed with 95% O_2_/5% CO_2_ and containing (in mM) 130 NaCl, 2.5 KCl, 2.0 CaCl_2_, 1.2 MgSO_4_, 1.25 KH_2_PO_4_, 25 NaHCO_3_ and 10 dextrose, and intracellular solution containing 135 K-gluconate, 2.0 MgCl_2_, 4.0 Na_2_-ATP, 0.4 Na-GTP, 10 Na-phosphocreatine and 10 HEPES adjusted to pH 7.3 with KOH. Cells were selected based upon neuron-like morphology and GFP marker expression (*n* = 15 from triplicate preparations). Recordings were conducted in both current-clamp and voltage-clamp modes with cell potential adjusted to −70 mV using Axon Instruments Multiclamp 700B amplifier, Digidata 1,440 analog-digital converter and pClamp 10.2 software. Input resistance was measured with −20 pA, 500 msec current steps and neuron-like excitability (action potential firing) was tested using 500 msec current steps ranging from −50 to +100 pA in current-clamp. Sodium currents and spontaneous excitatory postsynaptic currents (sEPSCs) were measured in voltage-clamp. sEPSCs were detected and measured offline using Minianalysis software with threshold amplitude set to 7.5 pA to avoid false positives from noise. Datasets of three 1-min recording epochs before, during and after CNQX per cell (*n* = 4) were analyzed by 1-way ANOVA followed by Tukey’s multiple comparisons test.

### Viral-vector preparation

3rd generation (self-inactivating) VSV-G pseudotyped Murine Moloney Leukemia Virus vector were produced by transfection of packaging (Gag/Pol, VSV-G) and vector plasmids into 293 T cells with the retroviral vectors collected into the cell culture supernatant or into Opti-MEM medium (serum free) for concentration by ultracentrifugation (Tiscornia et al., 2006). All vector transgenes were driven by the CAG promoter and co-expressed eGFP or dsRed under an internal ribosomal entry site (IRES) sequence. See Supplementary Table S1 for the origins of each retroviral vector plasmid.

### *In vivo* delivery and histology

Seven to eight week-old young adult male Long Evans Hooded rats were purchased from Harlan Sprague Dawley (Indiana, United States) for sole use in this study. Rats were housed two to three per cage under standard laboratory conditions with a light/dark cycle of 12 h with food and water *ad libitum*. All experiments were performed according to and approved by national and institutional guidelines (NIH, IACUC). Intracranial injection procedures adopted to minimize mechanical trauma to the cortex included careful drilling of a skull opening so that no mechanical compression of the dura or parenchyma occurs, use of a small gage needle to superficially create an opening in the dura for insertion of the delivery needle without contact with the cortical surface, and use of a specially designed 33G needle (Hamilton Neuros Syringe) to minimize needle track volume combined with slow delivery of viral volume. With these procedures, neuronal loss is confined to the volume of the 33G needle and hypertrophy of astrocytes is confined to the lining of the needle track and the cortical surface at the site of insertion. Rats were randomly assigned to viral delivery groups for the different transgenes. Viral injections were done for each transgene in groups of three to minimize wastage of the virus. Rats within each group were randomly assigned for collection of brains at the different analysis times. Rats were anesthetized with Isoflurane inhalant and injected with 3ul of concentrated retroviral CAG-eGFP (3 × 10^8^) or CAG-Neurogenin2-IRES-eGFP (3 × 10^8^) using a motorized stereotaxic injector (Stoelting) with delivery at 0.2 μL/min into either the right motor cortex or the right entorhinal cortex. The following coordinates were used: for motor cortex [from Bregma (mm): nosepiece −0.3; A/P: +0.5; M/L: −2.5; D/V(from dura): −2.0 raised 0.1 every minute] and for entorhinal cortex [from Bregma (mm): nosepiece: −7.0; A/P: ±9.2; M/L: −5.0; D/V(from dura): −4.5 raised 0.1 every 1 m 30 s].

Brains were collected at 1 and 2–3 weeks post injection following transcardial perfusion with 0.9% saline wash, followed by 4% paraformaldehyde (PFA) in phosphate buffer, then post-fixed in 4% PFA overnight at 4°C, equilibrated in 30% sucrose, and sectioned at 40 μm on a freezing stage microtome (Leica). Using unstained wet-mounted sections, all brains were screened for the detection of native GFP expression; brains that did not contain fluorescent cells or brains where the injection reached the corpus callosum or otherwise were not entirely within the cortex were not included in the study. Final group sizes were GFP (27) and Ngn2 (27). For immunofluorescence staining, net inserts (Corning Costar) were used to transfer free-floating sections between solutions and minimize handling. To remove cryoprotectant, all sections were rinsed multiple times in TBS followed by 3 h block in TBS++. For multiple immunostaining, sections were incubated for 72 h (at 4°C on a shaker table) with primary antibody diluted in TBS+. Primary antibodies and dilutions used are listed in Supplementary Table S2. Following two 1 h blocks with TBS++, sections were incubated for 48 h with Alexa Fluor 488 (Molecular Probes), Cy3, or Cy5 (Jackson Immunoresearch) diluted in TBS+ (1:500) for 2 h at room temperature. Following two 15-min rinses with TBS, all sections were mounted onto slides, cover slipped with PVA-DABCO mounting media, and stored at 4°C in the dark. Negative controls lacking primary antibodies were run along samples. The use of different reporter genes in some cases prevented the blinding of investigators to group identity.

### Microscopy and imaging

Confocal images were acquired using an Olympus DSU, Olympus Fluoview XV, Fluoview 500, or Leica SP8 resonance confocal microscope. Appropriate apertures and Nyquist sampling were used to acquire optimal resolution confocal stacks. Signal intensity was set objectively against a signal distribution histogram and with reference to positive and negative imaging controls to ensure an appropriate acquisition without oversaturation. Three-dimensional colocalization of signal was performed by generation of orthogonal views or through rendered three-dimensional visualization with rotation and orthogonal sectioning of the image volume using NeuroLucida software (MBF Bioscience, Inc.). Figures were composed in Adobe Photoshop with minimal adjustment to normalize signal distribution between panels and images were not otherwise manipulated.

### Quantitative stereology

The Navigator feature of the Leica LASX software was used to acquire a three-dimensional virtual tissue section of all sections containing GFP-positive cells. Imaging was performed on a Leica SP8 resonance confocal microscope using the 25 × 0.95 NA water immersion lens with a z-axis sampling interval of 1 μm to generate a focal series throughout the entire section thickness. All image stacks were stitched together to generate a virtual section. The series of virtual sections for each site of gene delivery were sampled using the StereoInvestigator software (MBF Bioscience, Inc.) to implement Optical Fractionator sampling to produce an estimate of total cell number (Peterson, 2004; Peterson, 2014). The sampling parameters used were: section series- every third section; area fraction 65 × 65 μm counting frame and a 125 × 125 μm sampling grid; optical disector height of 12 μm with a mean section thickness of 24.6 (±1.04) μm. This sampling density generated a mean coefficient of error (CE) value of 0.16 (±0.02). Cells were counted as they first came into focus using the optical disector counting rules and scored for single or multiple expression of fluorescent labeling. Data was summarized and analyzed using Prism 9.4 software (GraphPad, Inc.) with an initial ANOVA followed by a Bonferroni *post-hoc* test for significance with significance accepted at *p* ≤ 0.05.

## Results

### Characterization of adult-derived rat cortical OPCs

**Figure 1:**
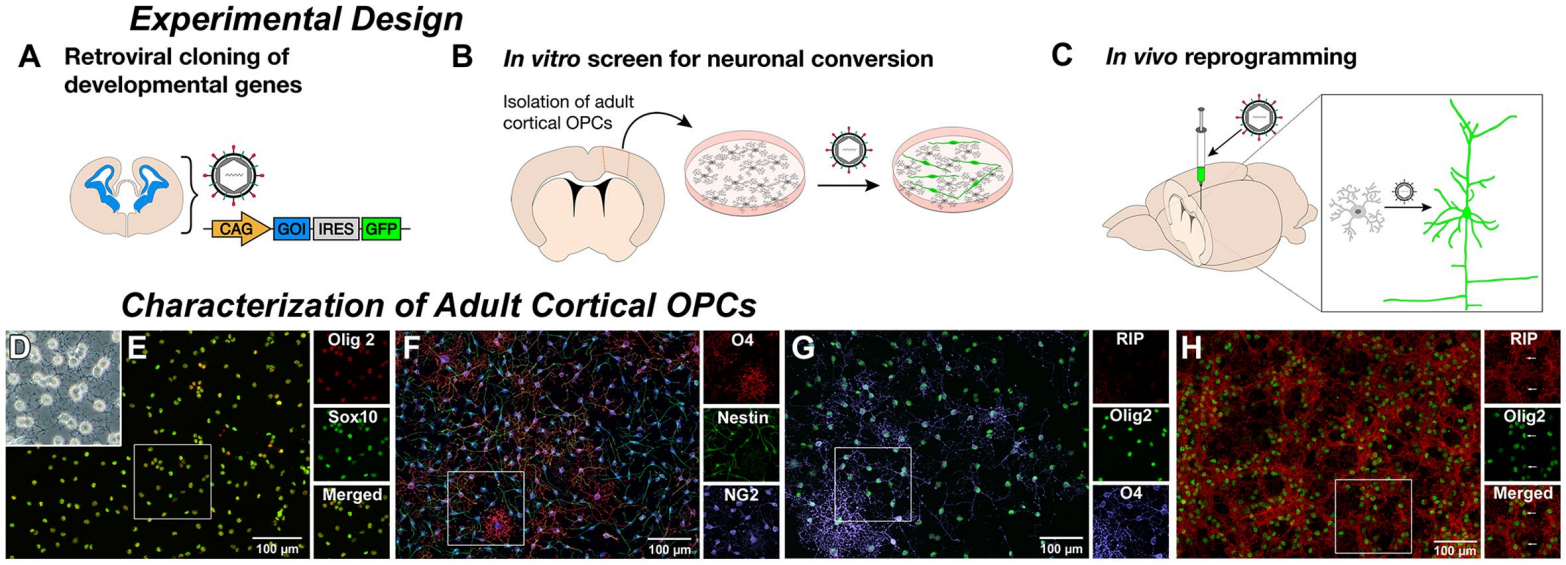
Derivation of adult rat cortical oligodendrocyte progenitor cells (OPCs). Experimental Design: **(A)** Genes encoding relevant developmental transcription factors were cloned into a retroviral vector with a fluorescent reporter gene. **(B)** Adult rat cortical gray matter was dissociated and O4-positive oligodendrocyte progenitor cells (OPCs) were selected, cultured, and transduced by retroviral delivery. β-III-tubulin expression of reporter-labeled cells served as a readout of neuronal induction. **(C)** Retrovirus containing successful constructs is then delivered to the cortex of a naïve animal to infect resident proliferating OPCs and induce neuronal reprogramming. Characterization of Adult Cortical OPCs: **(D)** Cultured O4-selected phase-bright multipolar cells with morphology consistent with an OPC identity. **(E)** Cells stain positive for pan-oligodendroglial lineage markers Olig2 and Sox10. **(F)** Isolated OPCs are NG2-positive and demonstrate a concomitant loss of nestin and increased O4 expression as cells progressed from early bipolar to later multipolar morphologies. **(G)** Under growth conditions, only the most elaborate O4-positive cells contained detectable staining for the premyelinating oligodendrocyte marker RIP. However, under oligodendrocyte differentiation media, **(H)** nearly all OPCs matured into RIP-positive oligodendrocytes, while maintaining expression of Olig2.

To prepare for future studies of neurogenic engineering in rat models of injury and dysfunction, we isolated and cultured adult rat cortical OPCs for use as an *in vitro* assay to identify successful transcription factors for subsequent *in vivo* cortical delivery to induce neurons from resident non-reactive OPCs (Figures 1A–C). To screen developmentally relevant pro-neuronal transcription factors (Imayoshi and Kageyama, 2014) (Supplementary Table S1), adult OPCs were isolated from cortical gray matter to replicate the target cell population for *in vivo* reprogramming. OPCs were selected for expression of the O4 antigen (Dincman et al., 2012) from dissociated adult rat neocortical gray matter, and could be cultured with the ability to expand, passage, and cryopreserve cells (Figures 1B,D). All adult cortical-OPCs expressed pan-oligodendroglial markers, Olig2 and Sox10 (Nishiyama et al., 2009) (Figure 1E) and NG2 (Figure 1F), but displayed maturational heterogeneity within the OPC lineage with a concomitant loss of nestin and increased O4 expression as cells progressed from early bipolar to later multipolar morphologies (Figure 1F). Under OPC growth conditions, most cells retained an OPC phenotype and the premyelinating oligodendrocyte marker RIP was rarely observed (Figure 1G). However, under oligodendrocyte differentiation conditions, RIP became strongly expressed (Figure 1H) confirming that the isolated OPCs were indeed within the oligodendroglial lineage and could proceed to terminal differentiation as oligodendrocytes with appropriate instruction.

### *In vitro* neuronal reprogramming

**Figure 2:**
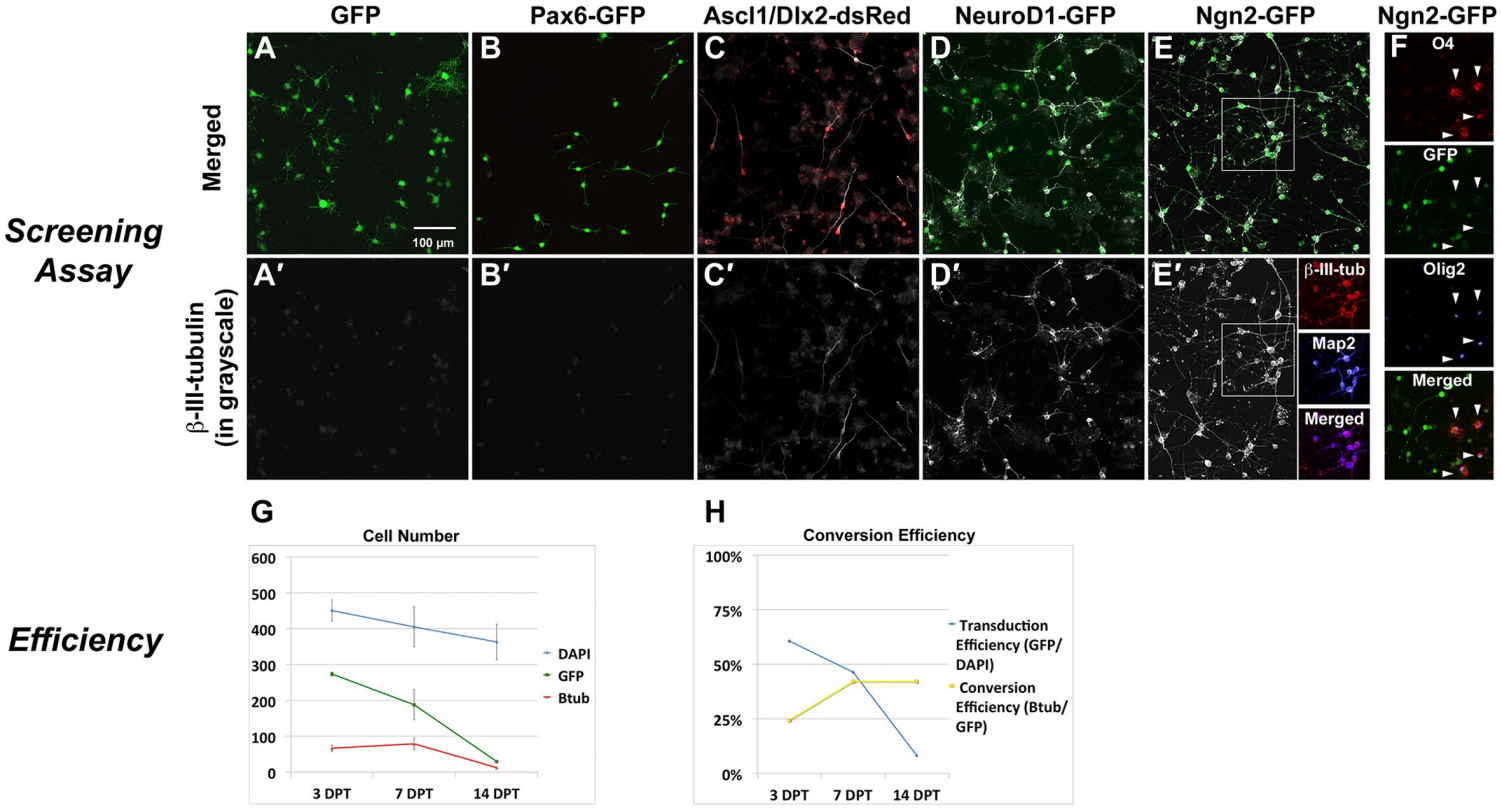
*In vitro* screen for reprogramming to phenotypic neurons. Screening Assay: OPC cultures received retroviral vectors for neurogenic transcription factors and were evaluated for neuronal induction based upon expression of the early neuronal marker β-III-tubulin. **(A)** GFP alone or **(B)** Pax6-GFP transduced OPCs but did not induce β-III-tubulin. Co-delivery of **(C)** Ascl1-dsRed and Dlx2-dsRed or **(D)** NeuroD1-GFP did induce β-III-tubulin. **(E)** Ngn2-GFP strongly induced β-III-tubulin, and induced neurons also expressed Map2. **(F)** Only non-transduced OPCs (non-GFP cells) continue to express Olig2 or O4 (arrowheads), indicating successful lineage respecification by Ngn2. Efficiency of Ngn2-induction: **(G)** The total number of cells in culture (DAPI) was reduced by about 20% over the course of the screening assay (14 days). Not all cells were transduced and the number of GFP-positive cells declined by about 90% over the course of the assay. Ngn2 successfully induced neurons, but the number of β-III-tubulin cells decreased by 80% by day 14. At all times, nearly 100% of β-III-tubulin cells were GFP-positive. Thus, loss of GFP positive cells occurred mainly in the portion that did not successfully convert into neurons. Values are mean ± standard deviation. **(H)** When stated in terms of efficiency, retroviral-Ngn2 transduced 60% of the cells and induced β-III-tubulin in 25% of those cells (i.e., 15% of total cells). Thus, over time, as the number of GFP cells declined, the apparent conversion efficiency increased.

Delivery of neurogenic transcription factors (Supplementary Table S1) cloned into retroviral vectors resulted in robust expression of the neuronal lineage marker ß-III-tubulin by 7 days (Figures 2A–E) following delivery of the single factors Neurogenin2 (Ngn2; 50% efficiency) or NeuroD1 (50% efficiency), and to a lesser extent with the combined delivery of Ascl1 and Dlx2 (15% efficiency), but not in any of the other combinations, including GFP-reporter control (0%) and Pax6 (0%). Ngn2-induced neurons (Ngn2-iNs) expressed ß-III-tubulin as early as 3 days post transduction (dpt) and expressed Map2 (Figure 2E′). Non-infected cells continued to express Olig2 and O4, while these were downregulated in Ngn2-infected cells (Figure 2F). Initial transduction efficiency was approximately 60% and of those GFP-expressing cells, neuronal conversion efficiency was approximately 25% (i.e., 15% of total cells; Figures 2G,H).

**Figure 3:**
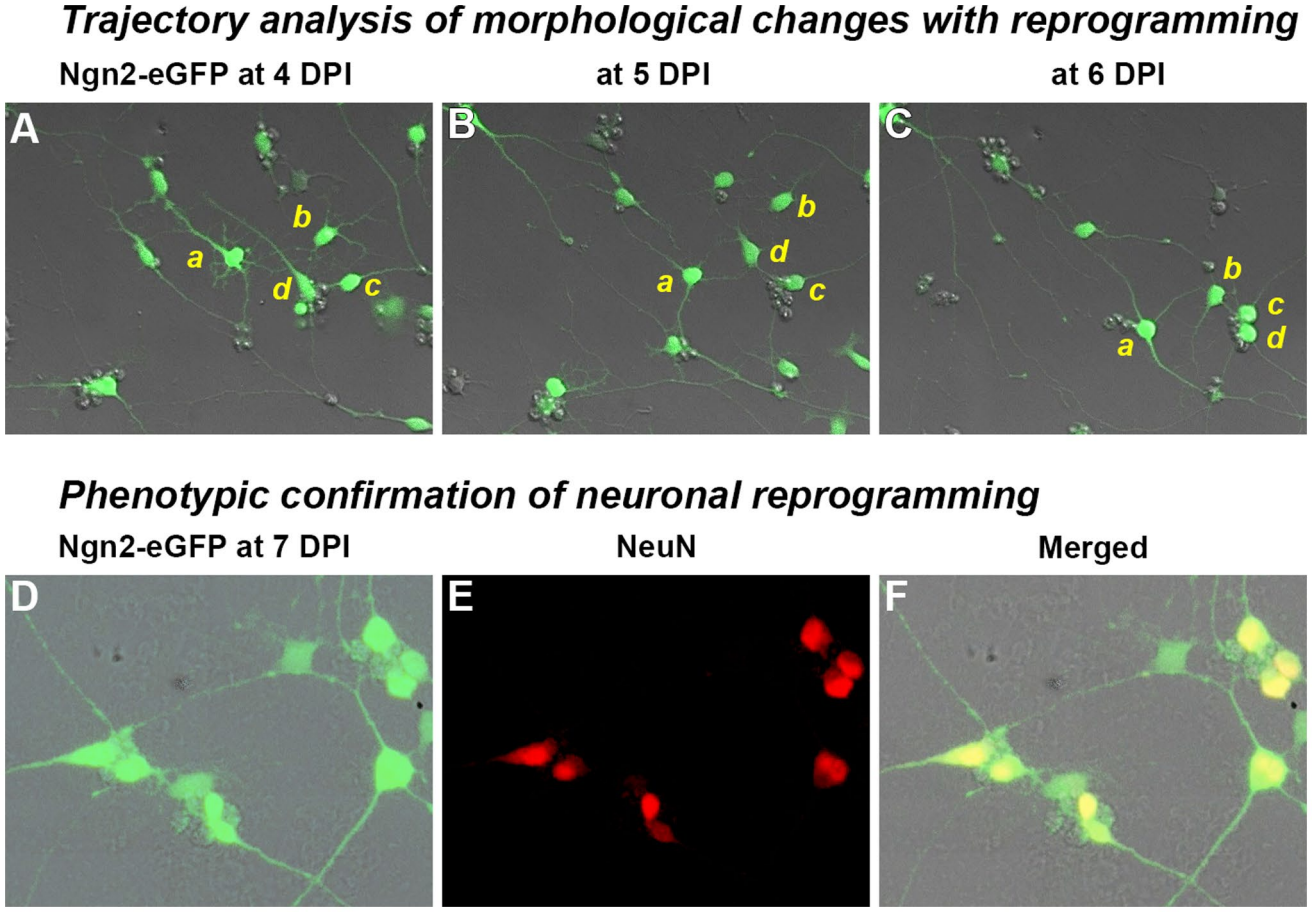
Trajectory analysis of Ngn2-expressing OPCs during the process of neuronal reprogramming. Trajectory analysis: Following retroviral delivery of Ngn2-eGFP (0 days post-infection or DPI), OPC cultures were observed by time-lapse imaging. A video summarizing the changes with neuronal reprogramming is included as Supplementary Material and can also be found at https://doi.org/10.5281/zenodo.8189544. **(A–C)** Four cells (*a*–*d*) are shown at 24 h intervals as they move, change morphology, and remodel their neurite extensions (see video for a dynamic view). Cells *a* and *b* exhibit a multipolar morphology at 4 DPI, while cells *c* and *d* are already showing more bipolar processes at this time. By 6 DPI, all four cells exhibit rounded cell bodies and distinct neurite processes that connect with neighboring induced neurons. Neuronal phenotype confirmation: **(D–F)** Cultures were fixed and immunostained at 7 DPI, revealing that nearly all GFP-positive cells expressed the neuronal marker, NeuN. Morphological progression and NeuN-positive cells were not observed in control cultures receiving retroviral eGFP-only delivery.

To confirm lineage progression from OPCs to induced neurons, we performed time-lapse imaging over 70 h from four to 6 days post-infection and conducted a trajectory analysis on the lineage progression of individual cells (Figure 3). Single frame images from each day during the imaging period (Figures 3A–C) illustrate morphological progression from multipolar short processes of OPCs to the eventual long processes typical of neuronal morphology that contact neighboring cells. The dynamic nature of this interaction can be seen in the Supplementary Video S1 (https://doi.org/10.5281/zenodo.8189544). Immunostaining immediately following confirmed the expression in these cultures of the mature neuronal marker, NeuN (Figures 3D–F).

**Figure 4:**
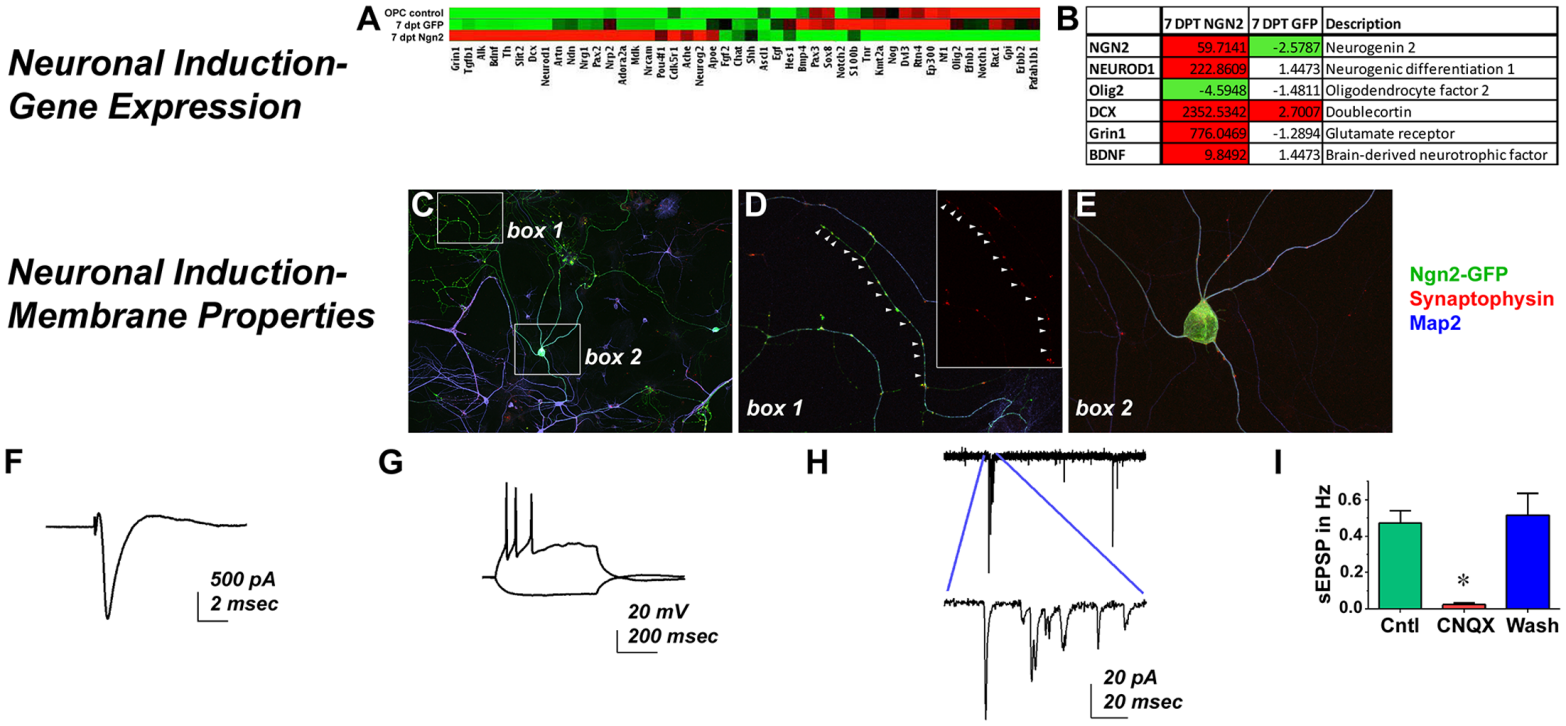
Ngn2 Expression Reprograms OPCs into Functional Neurons. Neuronal Induction-Gene Expression: **(A)** Control GFP-only transduced cells exhibited a largely similar gene expression profile to OPCs in a neurogenesis pathway PCR array, while Ngn2-induced neurons demonstrate distinct gene expression changes. **(B)** There was greater than a 4-fold expression increase in Ngn2-induced neurons for selected genes relevant to neuronal lineage, including NeuroD1 and DCX. Olig2, a pan-oligodendroglial lineage marker was actually reduced by more than 4-fold. Neuronal Induction-Membrane Properties: **(C)** Ngn2-GFP induced neurons (green) co-cultured with neonatal primary rat cortical neurons elaborate Map2-positive processes (blue). Primary neurons stain only with Map2. **(D)** There were extensive synaptophysin-positive contacts (red) on Ngn2-GFP dendrites. Inset shows synaptophysin staining alone. **(E)** Synaptic contacts were also found on dendrites near the soma and frequently on the soma itself. *In vitro* patch clamp recordings demonstrate **(F)** strong sodium currents **(G)** repetitive firing of action potentials upon depolarizing injection, and **(H)** spontaneous excitatory post-synaptic current activity that is **(I)** largely blocked by the glutamatergic AMPA receptor antagonist CNQX, indicative of synaptic input. Values are mean ± SEM, *F* (2, 6) = 23.28, *p* = 0.0015.

As an assessment of neuronal function following Ngn2 delivery, a neurogenesis-themed PCR array found that GFP-control vector largely matched the expression profile of OPCs while Ngn2-iNs upregulated neuronal genes (ie. DCX, Grin1) and the pro-neuronal transcription factor NeuroD1, but downregulated Olig2 by 7 dpt (Figures 4A,B). When co-cultured with P1 rat cortical neurons, Ngn2-iNs developed elaborate dendritic processes with punctate synaptophysin-positive contacts (Figures 4C–E), showed strong neuron-like voltage gated sodium current (Figure 4F), generated action potentials upon depolarization (Figure 4F), and displayed spontaneous excitatory post-synaptic currents that were inhibited by the glutamatergic AMPA receptor blocker, CNQX (Figures 4I,J). Thus, in addition to showing morphological and phenotypic features consistent with a neuronal identity, Ngn2-iNs evidence a similarity in transcriptional profile. Furthermore, Ngn2-iNs exhibited functional contacts and neuronal membrane properties consistent with a glutamatergic phenotype.

### Specificity of *in vivo* retroviral delivery for cortical OPCs

**Figure 5:**
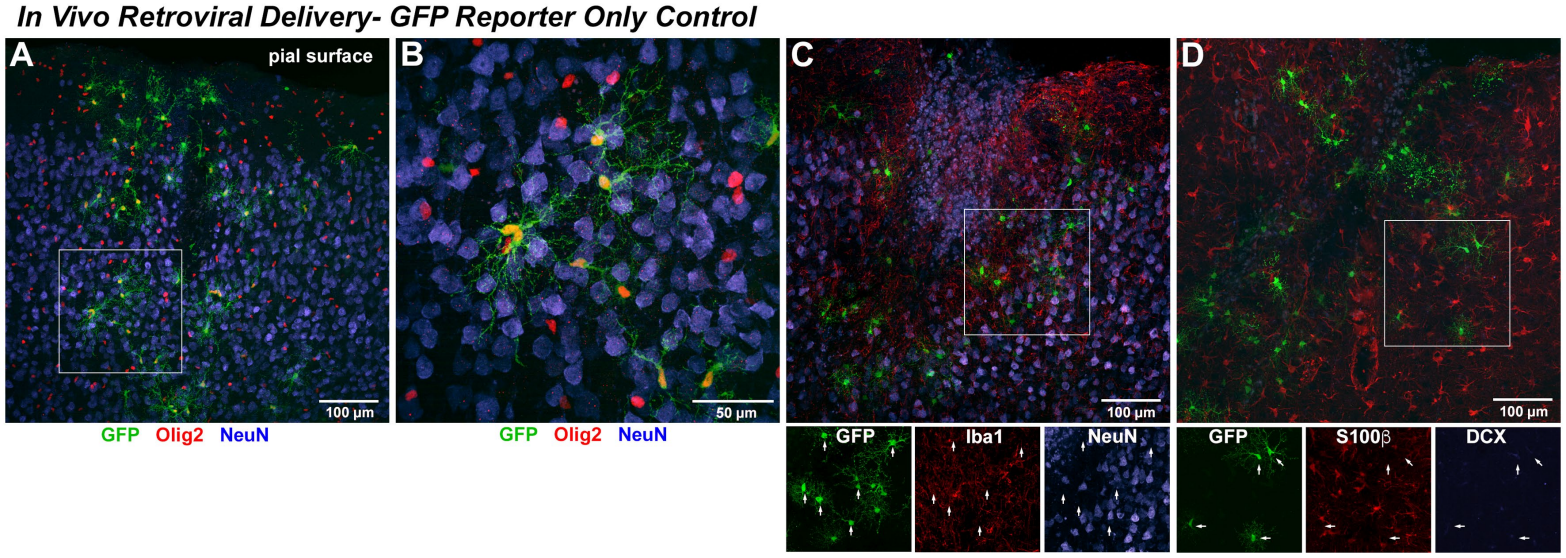
*In vivo* retroviral delivery of GFP reporter-only control infects proliferating OPCs but does not induce any detectable neuronal phenotype. *In Vivo* Retroviral Delivery-GFP Reporter Only Control: As retrovirus infects only dividing cells and the vast majority of proliferating cells in the naïve brain are OPCs, it was expected that this population would be targeted by retroviral delivery. By 7 days following *in vivo* cortical delivery of retroviral-GFP control vector, a distinct population of transduced cells was observed. **(A)** GFP-positive cells with a complex branching morphology consistent with NG2 Glia were uniformly distributed. A systematic, quantitative examination of coexpression with GFP revealed that 99.7% of GFP-positive cells observed in the control condition were positive for the OPC marker Olig2 (red) or Sox10 (not shown) by 7 DPI. **(B)** GFP-positive cells were distributed amongst mature neurons (NeuN, blue) and were often closely associated with, but distinct from, neurons. NeuN-positive cells were never observed to coexpress GFP. To verify that no GFP-positive cells were other neural cell types, adjacent sections were stained for **(C)** Iba1 (red) to detect microglia or **(D)** S100β (red) to detect astrocytes. In no case were GFP-positive microglia or astrocytes observed, as would be predicted for a naïve brain without previous injury where these cells are not actively proliferating. Staining for the early neuronal marker DCX revealed an absence of DCX expression in GFP-only positive cells (DCX detection in the hippocampal dentate gyrus within the same section was used as a positive control for validation of imaging parameters).

We next asked if *in vivo* retroviral delivery of Ngn2 could also reprogram non-reactive OPCs within the naive rat cerebral cortex into neurons. As predicted from the fact that OPCs are the primary proliferative cells in the naive adult cortex, we found that GFP-reporter labeled cells expressed the pan-oligodendroglial marker, Olig2, with characteristic OPC morphology (Figures 5A,B). Based upon viral delivery parameters, each injection infected a fraction of Olig2-positive cells in this cortical volume in the rat, reflecting the proportion of OPCs undergoing cell cycle at the time of retroviral availability. With GFP-reporter only delivery, infected cells rarely express markers for immature (DCX) neurons and then only weakly. However, GFP-positive cells did not co-express markers for mature (NeuN) neurons, mature astrocytes (S100ß), or microglia (Iba1) at 7 days post injection (dpi; Figures 5A–D). By using detection of soma-localized S100ß for astrocytes, we were able to unambiguously exclude co-localization of astrocytes with retroviral GFP-reporter (Figure 5D).

Olig2 is a transcription factor expressed throughout the oligodendrocyte lineage apart from mature oligodendrocytes, while Sox10 is a transcription factor expressed from early OPCs to terminal oligodendrocytes. OPCs begin to downregulate expression of NG2 as they progress to becoming mature oligodendrocytes. To further characterize the proliferating cell population targeted by retroviral gene delivery, which infects only dividing cells, we analyzed control eGFP-only *in vivo* retroviral delivery at the 7 day post-injection interval and found that 85.9% (±2.6%) were Sox10+/NG2+ OPCs and the remaining 13.8% (±2.7%) were Sox10+/NG2− oligodendrocytes. Thus, 99.7% (±0.03%) of GFP-expressing cells are OPCs or OPCs progressing toward terminal differentiation. As only proliferating OPCs were targeted by retroviral delivery, only 23.4% (±5.8%) of OPCs present at the point of retroviral delivery expressed GFP.

### *In vivo* cortical neuronal reprogramming by Ngn2

**Figure 6:**
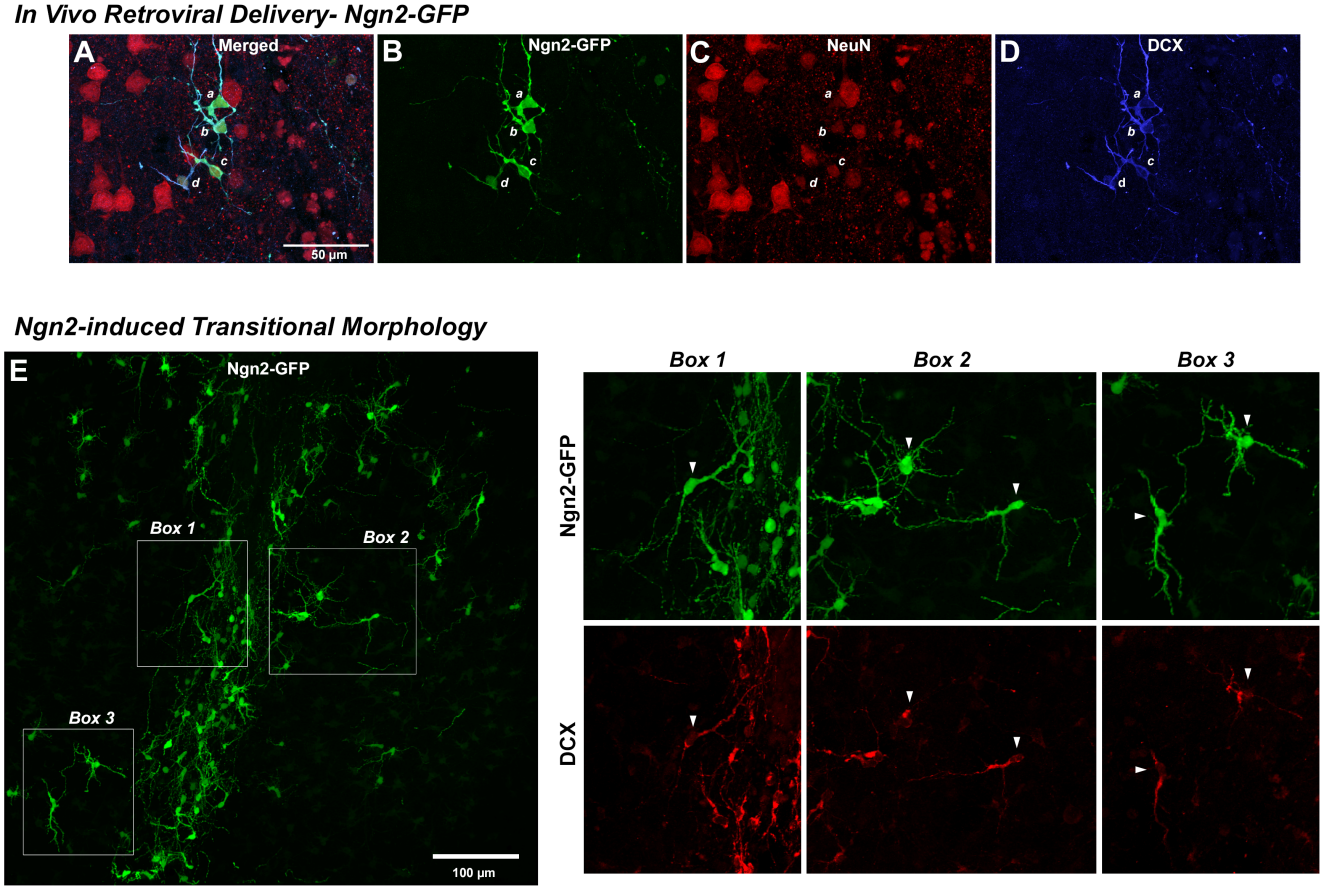
Reprogramming *in vivo* OPCs by Ngn2 induces neuronal lineage commitment. *In Vivo* Retroviral Delivery-Ngn2-GFP: Within 7 days, delivery of Ngn2-GFP resulted in a population of cells (four of which are identified by *a*, *b*, *c*, and *d*) expressing the GFP reporter **(A,B)**. Ngn2-induction also initiated neuronal lineage commitment evidenced by their co-labeling with DCX **(D)** and in some cells there may be some weak expression of NeuN **(C)**, indicating a further progression toward neuronal maturation. Ngn2-induced Transitional Morphology: **(E)** Ngn2-GFP-positive cells exhibited elaborate process extension with the adoption of defined cell polarity, similar to the dynamic morphological transition observed *in vitro* in Figure 4. Cells indicated in the boxed regions are shown at right with their co-expression of DCX. The elaboration of polarized cell morphology, extensive varicose process extension, weak NeuN expression, and discontinuous DCX staining suggests adoption of early neuronal lineage with eventual transition from DCX-positive neuroblasts to NeuN-positive neurons with a mature morphology. While some very weak DCX staining was observed at 7 days following retroviral GFP control delivery, this distinct DCX-positive phenotype and polarized cell morphology was only observed following retroviral Ngn2-delivery.

Retroviral delivery of Ngn2 induced DCX expression in nearly all Ngn2-GFP-positive cells by 7 days (Figures 6A–D). Ngn2-iNs began to express NeuN, although at weaker levels than pre-existing neurons by 7 dpi. Given the robust process extension and morphological remodeling observed of Ngn2-iNs *in vitro* at a similar time (Figure 3 and Supplementary Video S1), it is perhaps not surprising that *in vivo* Ngn2-iNs exhibited substantial process extension and complexity with cell polarity suggesting a transitional immature neuronal morphology (Figure 6E). These Ngn2-iNs also expressed the early neuronal lineage marker DCX, confirming their progression through a neuronal lineage (Figure 6E).

**Figure 7:**
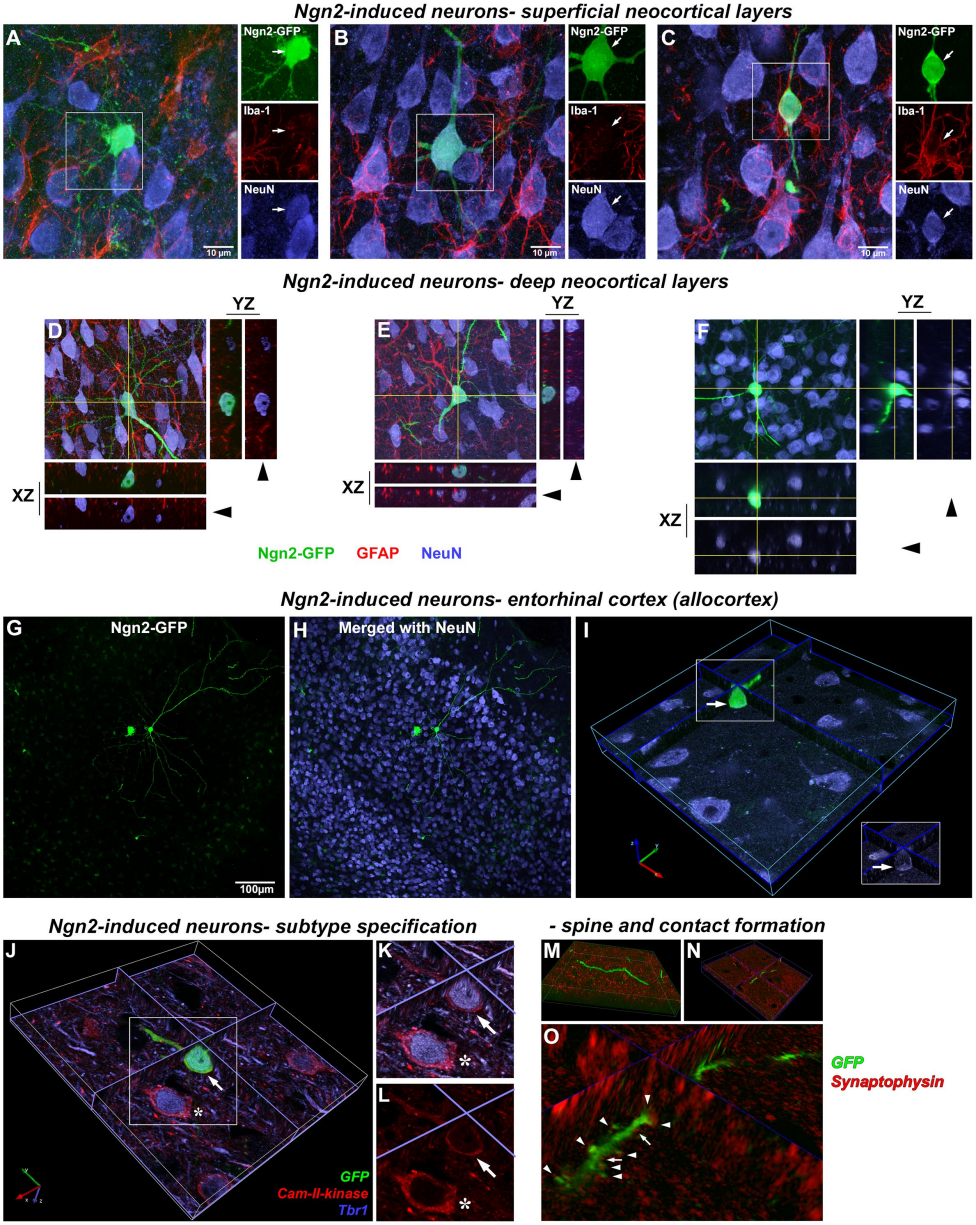
Morphologically mature neurons induced by *in vivo* Ngn2-reprogramming. Superficial cortical layers: by 3 weeks, Ngn2-GFP induced neurons showed an advancement in neuronal lineage commitment as evidenced by a more mature and cytoarchitecturally appropriate neuronal morphology. Superficial cortical layers contained induced neurons exhibiting a continuum of morphological maturation, but also showing coexpression of the mature neuronal marker NeuN (blue). **(A)** Some Ngn2-GFP-positive cells evidenced less maturation by 3 weeks, exhibiting incomplete process polarity, varicosities in processes and a lack of dendritic spines, but still expressing NeuN as evidence of neuronal lineage commitment. However, Ngn2-induced neurons did not co-express Iba1 (red), indicating the absence of non-specific uptake of GFP by microglia. **(B)** Other Ngn2-induced neurons exhibited a distinctly mature neuronal morphology with dendritic branching and a descending axon that left the plane of section within 50 μm from the soma along with strong NeuN co-expression. **(C)** Yet other Ngn2-induced neurons exhibited a less complex morphology, but still strongly expresses NeuN. GFP signal outside of the boxed region belongs to other induced neurons and their processes that lay beyond the focal planes included in this image. Images are presented as a maximum projection of a number of focal planes to provide a three-dimensional representation. Deep cortical layers: Deeper cortical layers also contained Ngn2-induced newly-generated neurons with layer-appropriate cytoarchitecture including an Ngn2-induced neuron **(D)** with an inverted pyramidal morphology only seen in cortical layer V neurons. Orthogonal projections in the XZ and YZ planes at the level indicated by the yellow lines are included for each maximum projection image to validate coexpression of staining in three-dimensions. Confocal image stacks showing three examples of Ngn2-induced neurons **(D–F)** confirm these cells are NeuN positive (blue); arrowheads indicate removal of GFP-signal. Panels **(D,E)** also illustrate the absence of GFP coexpression in GFAP-positive astrocytes (red). In fact, no GFAP-positive astrocytes were observed to express GFP. Entorhinal cortex (Allocortex): In addition to the motor cortex, OPCs can be induced by retroviral Ngn2 delivery to convert to a neuronal lineage in other cortical regions. Retroviral delivery of Ngn2-GFP to the entorhinal cortex (allocortex) also induced new neurons by 3 weeks, including this example in entorhinal cortical layer III **(G,H)**. However not all infected cells survived the reprogramming instruction as shown by the dying cell to the left of a cytoarchitecturally appropriate new neuron. The successfully reprogrammed new neuron on the right also expresses NeuN **(I)** as evidenced by the three-dimensional image shown digitally sectioned at the planes indicated at the blue lines. The boxed region in inset validates the colocalization with NeuN following the removal of the GFP-signal overlay (arrow). Neuronal subtype specification: The subtype specification of GFP-positive Ngn2-induced new neurons (**J**; arrow) to a glutamatergic phenotype is shown by their co-expression of Tbr1 (blue) and Cam-II-kinase (red). This expression is identical to adjacent pre-existing neurons (asterisk). **(K)** Inset shows digital resectioning of a confocal image stack at the planes indicated at the blue lines with digital removal of the GFP signal (arrow) leaving the combined Tbr1 (blue) and Cam-II-kinase (red) signal. **(L)** Digital removal of the Tbr1 signal leaves only the Cam-II-kinase expression, cumulatively demonstrating the coexpression of these glutamatergic phenotype markers in the induced neuron. Dendritic spine and synaptic contact formation: Apical and basal dendrites of all newly-generated neurons contain abundant small spines **(M,N)** shown in different orientations from a confocal image stack. Three-dimensional rendering of dendrites and spines **(O)** reveals that both spines (arrowheads) and dendritic shafts (arrows) receive synaptophysin-positive contacts (red) indicating that preexisting axons are making synaptic contact with the newly-generated neurons.

As Ngn2 reprogramming *in vitro* also drove very high expression of NeuroD1 (Figures 4A,B), we next asked if *in vivo* Ngn2 reprogramming alone was sufficient to generate pyramidal neuronal morphology with glutamatergic subtype specification. By 3 weeks, Ngn2-iNs were observed in superficial layers of the cortex (Figures 7A–C) showing a continuum of progression toward a mature neuronal morphology with distinct dendritic and axonal processes and, in all cases, distinctly co-expressing the mature neuronal marker NeuN. In all cases, signal co-expression was assessed in three-dimensions to verify that signals overlap in the same cellular compartments. All neurons, including the Ngn2-iNs, were closely associated with Iba-1-positive microglia (Figures 7A–C), but no microglia were observed to co-express GFP, indicating the absence of non-specific GFP uptake.

Ngn2-iNs were also observed by 3 weeks in deep cortical layers (Figures 7D–F) that distinctly co-expressed NeuN and exhibited cytoarchitecturally appropriate pyramidal cortical neuron morphology, including primary and basal dendrites, dendritic arbor elaboration, and dendritic spines. Evaluation of GFAP-positive cells confirmed the absence of GFP expression in astrocytes. Separate Ngn2-GFP gene delivery to the phylogenetically older entorhinal cortex (allocortex) also resulted in the generation of induced neurons by 3 weeks (Figures 7G–I) demonstrating that OPCs in regions other than neocortex may be amenable to neuronal reprogramming.

*In vitro* data had demonstrated that Ngn2-iNs established functional glutamatergic contacts (Figure 4). To determine if Ngn2-iNs generated following *in vivo* gene delivery adopt a glutamatergic neuronal subtype specification, GFP-positive Ngn2-iNs were examined for co-expression of the cortical glutamatergic neuronal markers Tbr1 and Cam-II-kinase-α. Evaluation of three-dimensional imaging (Figures 7J–L) revealed that both markers were detected, indicating that rat cortical OPCs had been reprogrammed by Ngn2 expression alone in the absence of prior injury into a glutamatergic neuronal subtype. We next evaluated dendrites and dendritic spines on Ngn2-iNs (Figures 7M–O) and identified synaptophysin-positive contacts on both GFP-positive dendrites and dendritic spines indicating the presence of morphological contacts with the pre-existing cortical circuitry.

### Stereological quantification of neuronal reprogramming

**Figure 8:**
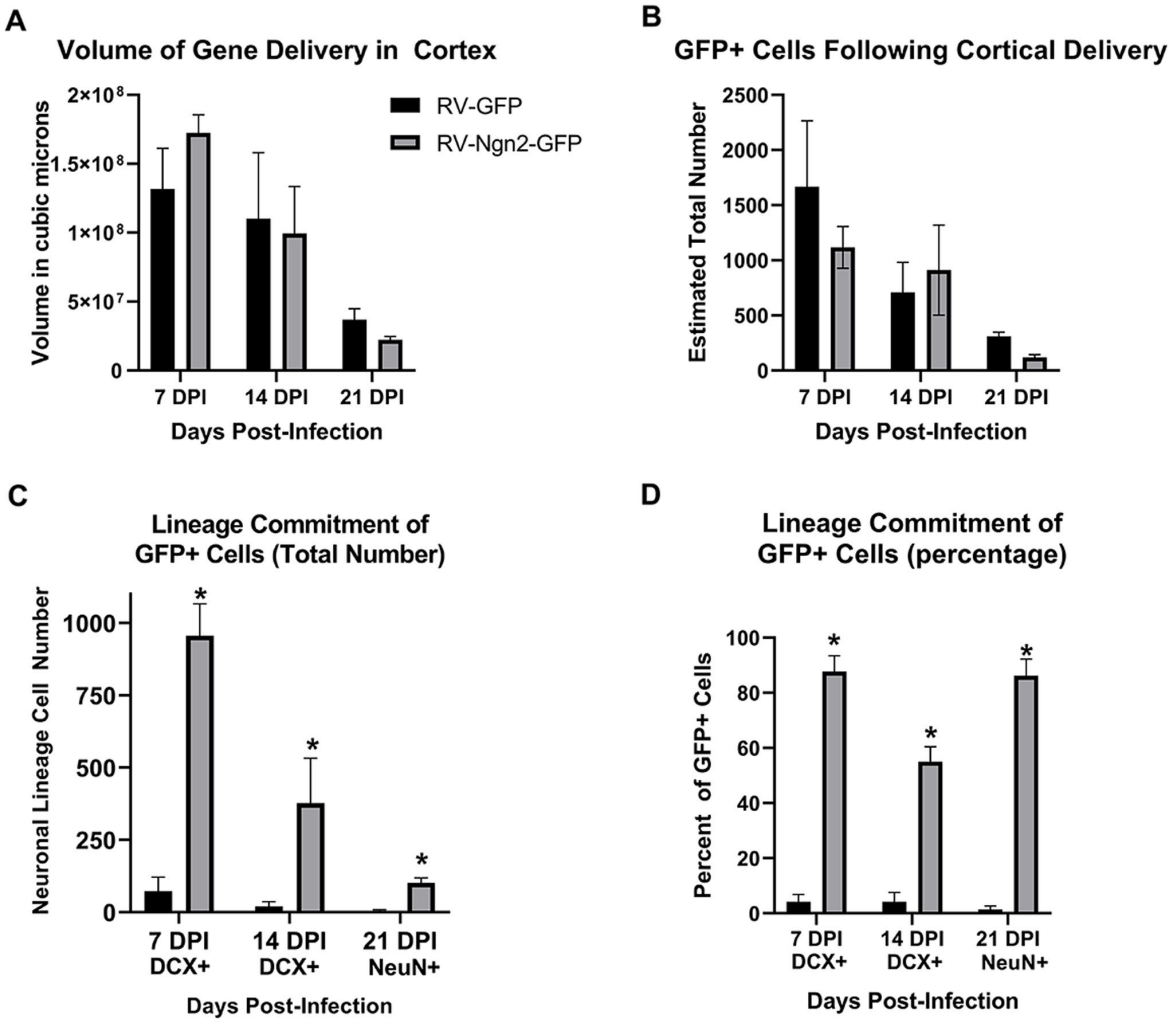
Stereological quantitation of OPC infection by retroviral delivery and subsequent neuronal reprogramming outcomes. **(A)** The volume of cortex containing GFP-positive cells following retroviral delivery did not differ between the control, GFP-only vector, and the experimental, Ngn2-GFP vector, at any time point. However, the reduction in cell survival over time is reflected in the declining volume occupied by GFP-positive cells over time. **(B)** The determination of total number of GFP-positive cells likewise showed no difference between GFP-only and Ngn2-GFP infection at any time point, although a decline in total number is evident over time. **(C)** GFP-positive cells were quantified for coexpression of the early neuronal lineage marker DCX at 7 days or 14 days, or coexpression with the mature neuronal lineage marker NeuN at 21 days. The GFP-only control group contained only few cells expressing some weak DCX labeling at early time points and only a single cell was observed to weakly express NeuN at 21 days. The number of GFP-positive cells expressing neuronal markers in the Ngn2-GFP group is significantly higher at all time points. **(D)** When expressed as a percentage of GFP-positive cells for each condition at each time point, retroviral delivery of Ngn2 resulted in a high percentage of all remaining GFP-positive cells being induced neurons at all time points.

We performed stereological quantitation (Figure 8) to assess the number of GFP-positive cells in total for neocortical retroviral gene delivery of both control GFP-only and Ngn2-GFP at three time points (7, 14, and 21 days post-delivery). Cells were counted based upon their expression of GFP alone, or coexpression with the early neuronal lineage marker DCX (7 and 14 day groups) or the mature neuronal marker NeuN (21 day group). The volume of cortex occupied by GFP-positive cells did not differ between control and Ngn2 induction at any time point but did reveal a decline over time (Figure 8A). The basis for this volumetric decline can be seen by the estimation of total GFP-positive cell number (Figure 8B). Although there was no difference at any time between control GFP-only and Ngn2-GFP conditions, overall the populations of cells declined. The extent of initial gene delivery distribution within the cortex varies from subject to subject. This variable, and likely other nuances of intracerebral delivery, contributed to substantial variance with the result that statistical evaluation by ANOVA did not produce adequate significance to proceed with subsequent between group tests.

Estimation of the number of GFP-positive cells that showed neuronal lineage commitment (Figure 8C) demonstrated that the Ngn2-induced groups significantly generated induced neurons compared to the control GFP-only group. In fact, the control GFP-only group had few GFP-positive cells that were weakly DCX-positive. Despite the weakness of staining, these cells were included in the counts for 7 and 14 day groups. In the 21 day group for the GFP-only control condition, no GFP-positive cells were detected that co-expressed NeuN apart from a single GFP-positive cell that showed weak positivity for NeuN. This cell was included in the count, which also enabled statistical testing to be performed for the 21 day group. When evaluated for neuronal lineage commitment as a percentage of GFP-positive cells (Figure 8D), the weakly DCX-positive cells in the control GFP-only condition represent less than 4% of GFP-positive cells in the 14 day group. The single weakly NeuN positive cell represents less than 1% of GFP-positive cells in the 21 day group. In contrast, neuronal lineage commitment is significantly higher in the Ngn2-induced condition, with more than 85% of GFP-positive cells identified as neurons by 21 days.

## Discussion

In this report, we demonstrate that delivery of a single transcription factor, Ngn2, can reprogram resident cortical OPCs into morphologically mature, subtype-specific pyramidal neurons in multiple rat cortical regions (including neocortex and allocortex) that are otherwise entirely devoid of neurogenesis. The relatively rapid expression of mature neuronal features we observed *in vitro* and *in vivo* may be explained by the reports that forced Ngn2 expression produces rapid expression of many mature neuronal transcriptional programs (Masserdotti et al., 2015; Kempf et al., 2021). Although possibly still in the process of maturing, these Ngn2-iNs receive multiple contacts on their dendrites and dendritic spines from pre-existing neurons. Neuronal electrophysiological properties were evident in Ngn2-iNs by 1 week *in vitro*. By using retroviral delivery of the reprogramming factor, we avoided targeting preexisting neurons, as has been a possibility with AAV delivery (Calzolari and Berninger, 2021; Wang et al., 2021; Chen et al., 2022; Cooper and Berninger, 2022). However, this experimental design also precluded employing the strategy of prior injury, reportedly important to achieve reprogramming (Grande et al., 2013; Guo et al., 2013; Heinrich et al., 2014; Torper and Gotz, 2017; Lentini et al., 2021), as this would have induced proliferation in a broader population of cells and prevented our goal of targeting the OPC population. Nevertheless, retroviral delivery of Ngn2 to proliferating OPCs resulted in their reprogramming into neurons, suggesting that the cortical environment of the adult rat can continue to foster neuronal maturation.

### Specificity of reprogramming factor delivery

Retroviral transgene expression is restricted to cells undergoing cell division and thus also serves as a marker of cell cycle (Morshead and van der Kooy, 1992; Torper and Gotz, 2017). We therefore anticipated that retroviral delivery of reprograming factors to the naïve cortex would primarily target OPCs (Dawson et al., 2003), while also serving as a control for the inability to infect pre-existing post mitotic neurons. This straightforward delivery strategy also avoids potential false positive detection due to leaky expression of Cre under OPC-specific promoters that has been responsible for mistaken *in vivo* differentiation of OPCs to neurons (Richardson et al., 2011). Although a previous report (Guo et al., 2013) identified retroviral infection of astrocytes in mouse cortex, these cells only proliferate several days following an injury, and astrocyte proliferation may be misidentified and overstated due to the extent of GFAP upregulation (Burda and Sofroniew, 2014; Dimou and Gotz, 2014). This study design avoided any prior injury or activation of glial cells into a reactive state that would also stimulate proliferation in other cell types (Hampton et al., 2004), resulting in the absence of any retrovirally-delivered GFP detected in cells other than the constitutively proliferating OPC population. Thus although it is possible that occasional proliferating pericytes, endothelial cells, microglia, or astrocytes could potentially be included among infected cells, on a population basis, these other cell types were not detected to express GFP and the infected cell population was primarily OPCs, consistent with the non-reactive milieu at the time of viral infection. In control GFP-only conditions, we observed a portion of the GFP-expressing cells were Sox10-positive without NG2 expression. It is possible that some of these cells may have been oligodendrocytes proliferating at the time of retroviral delivery (Simon et al., 2011). The remaining population may have been OPCs at the time of retroviral delivery that subsequently downregulated NG2 as they progressed over the 7 days toward terminal differentiation as oligodendrocytes, a number and timeframe that is consistent with another report (Shimizu et al., 2020). Furthermore, we were able to exclude the participation of SVZ-derived neuroblasts as the source of our identified Ngn2-iNs based upon their absence in control GFP-only conditions and the identification of Ngn2-iNs in the entorhinal cortex, the most remote cortical region from the anterior SVZ.

### Ngn2 reprogramming induced progression through neuronal lineage

Neurons have distinct phenotypic subtypes and the generation of new generic neurons without these properties and without appropriate morphology may be of limited value for repair. Thus, our goal was to achieve reprogramming of naive OPCs to distinct neuronal subtypes with cytoarchitecturally appropriate neuronal morphology. An important validation to the authenticity of neuronal reprogramming is the progression from earlier to more mature neuronal phenotypes (Calzolari and Berninger, 2021). Both our *in vitro* and *in vivo* data demonstrated that expression of Ngn2 in OPCs resulted initially in a transition in morphology and the expression of early neuronal lineage commitment markers. By following the morphological trajectory of cultured rat OPCs expressing GFP, the transition to a neuronal morphology could be confirmed in individual cells and the expression of mature neuronal markers subsequently confirmed.

Following *in vivo* delivery, Ngn2-iNs begin to adopt more mature neuronal morphologies and express more mature phenotypic markers and evidence of synaptic contact with the pre-existing neuronal circuitry. Newly Ngn2-engineered neurons were detected in both more superficial and deep layers of the motor cortex (neocortex) and also the phylogenetically distinct entorhinal cortex (allocortex). The appropriate pyramidal neuron phenotype of Ngn2-iNs was confirmed by their expression of the cortical glutamatergic neuronal markers that matched adjacent pre-existing mature neurons and indicates phenotypic subtype specification, consistent with the *in vitro* functional data indicating glutamatergic specification. The adoption of cytoarchitecturally appropriate, location specific morphology also suggests competence to respond to remaining local environmental factors in establishing final maturation. For example, inverted pyramidal neurons comprise a small projection neuron subset confined to the deep cortical layers (Mendizabal-Zubiaga et al., 2007), and this morphology is appropriately induced in a subset of Ngn2-iNs in the deep layers. Similarly, entorhinal Ngn2-iNs adopt a location appropriate morphology. Thus, our ability to target OPCs almost exclusively by using retroviral gene delivery and the demonstrated progression from earlier to mature neuronal lineage validate that these are newly generated neurons and argue against the concerns that have been identified with some previous studies (Calzolari and Berninger, 2021; Wang et al., 2021; Chen et al., 2022; Cooper and Berninger, 2022).

### Authenticity of neuronal reprogramming

Apart from a single weakly NeuN-positive cell observed in one GFP-control subject at 21 days, no GFP-neurons were detected in control GFP-only injected animals. Nevertheless, the observation of morphologically appropriate neurons emerging by 21 days following retroviral delivery of Ngn2 led us to examine the possibility of a fusion event or other artifact, whereby GFP was simply transferred to a pre-existing neuron. Fusion events have been reported in neonatal brain, where it was mediated by infected microglia, but not in adult cortex (Ackman et al., 2006). We carefully examined Ngn2-iNs using high-resolution imaging and 3-dimensional rendering and found no cases of process fusion or double nuclei. Ngn2-iNs were contacted by Iba1-positive microglia, as were pre-existing neurons but in no cases were the Iba1 cells also GFP-positive. Furthermore, Ngn2-iNs passed through an immature, DCX-expressing neuronal state with advanced morphological complexity prior to the appropriate neuronal morphology seen later. In addition, Ngn2-iNs are not synchronous in their lineage progression and display a range of maturation, further arguing against transfer of GFP to pre-existing neurons.

### Efficiency of neuronal reprogramming

The reduction in number of infected cells prior to maturation suggests that only fully reprogrammed Ngn2-iNs survive the reprogramming experience (Gascon et al., 2016). This low survival rate is not entirely surprising as 50–80% of adult newborn dentate granule cells die in an environment that is normally supportive of adult neurogenesis (Dayer et al., 2003; Sandoval et al., 2011). Similar outcomes in efficiency of neuronal reprogramming have been linked to regional heterogeneity of astrocytes (Hu et al., 2019) and this may also be true of OPCs. The efficiency of neuronal reprogramming is consistent with that seen in our *in vitro* data and in other reports (Guo et al., 2013), suggesting the possible death of cells that fail to achieve full re-specification. Furthermore, we estimate that only some 25% of available OPCs were targeted using retroviral delivery of the reprogramming factor. Increasing the number of cells targeted and the reprogramming efficiency to generate more induced neurons will be a goal for future studies.

### Reprogramming capacity of the naïve rat cortex

There are two additional aspects that distinguish this report from most previous studies investigating cortical neuronal reprogramming. Previous studies in mouse cortex made the point that activation of astrocytes or glial progenitor cells by injury prior to delivery of reprograming factors was needed to achieve neuronal induction (Guo et al., 2013; Heinrich et al., 2014; Lentini et al., 2021). Using transcription factors alone for neuronal re-specification had not achieved this goal, suggesting that the prior injury or pathology was needed to generate a reactive or environmental state where the cells could be amenable to reprogramming (Grande et al., 2013; Guo et al., 2013; Heinrich et al., 2014; Torper and Gotz, 2017). In the present study, our retroviral targeting design avoided the establishment of an injury response state and provided insight as to the autonomous capacity of transcription factors to re-specify lineage (Guo et al., 2013; Heinrich et al., 2014). We reasoned these data could then also provide insight in regard to experimental or potential therapeutic modulation of local circuitry in disorders without a proximal injury response, such as psychiatric or addictive disorders (Southwell et al., 2014). This is not to say that an injury response after delivery of the reprogramming factor may not play a role in the process of lineage respecification. For example, some astrocytic hypertrophy was observed at the injection sites at 7 days post-injection and more severe cortical injury models note substantial proliferation of microglia and NG2-positive cells within 2–4 days (Hampton et al., 2004; Simon et al., 2011; von Streitberg et al., 2021). However, the data reported here demonstrate that neuronal reprogramming is possible without previously generating a reactive state in the target cell.

Another distinguishing feature of the present study is that the capacity for neuronal reprogramming is demonstrated in the naïve rat cortex, not the mouse cortex, which most studies to date have used. While mouse models are undeniably powerful research tools, many other important disease and behavior models have been developed using rats. Here, we demonstrate that cortical neuronal reprogramming is possible in naïve rat CNS, supporting the feasibility of extending studies of cell lineage specification into this important research tool.

## Conclusion

This study is primarily a proof of concept for developing OPC reprogramming for neural regeneration. While, there is some possibility that retroviral vectors could be developed for safe use in the clinic in the future, it is far more likely that therapeutic delivery would ultimately utilize a host of other vector systems with better safety profiles. However, complex retrovirus vectors are being used clinically (Jogalekar et al., 2022) and past incidence of simple retroviral vector oncogenesis (Hacein-Bey-Abina et al., 2008) has been linked to the oncogenic nature of the transgene itself and not to the vector alone (Woods et al., 2006). It should also be noted that the expression of differentiation factors should inhibit oncogenesis (Lacomme et al., 2012).

While further studies are needed to elucidate the mechanisms of neuronal lineage re-specification, increase the efficiency of reprogramming OPCs into neurons, and to evaluate the functional integration of newly induced neurons into existing neuronal circuitry, the present study demonstrates the feasibility of neuronal reprogramming in the naïve rat cortex. These data suggest that direct *in vivo* fate reprogramming of resident non-reactive OPCs may provide a potential avenue for repair in the adult brain, both for neuronal replacement to restore circuitry and conceivably for neuronal addition to modulate circuitry in neurological disorders.

## Data availability statement

The raw data supporting the conclusions of this article will be made available by the authors, without undue reservation.

## Ethics statement

The animal study was approved by Rosalind Franklin University Institutional Animal Care and Use Committee. The study was conducted in accordance with the local legislation and institutional requirements.

## Author contributions

SB, AM, RP, and MT isolated cortical progenitor cells and performed and analyzed *in vitro* studies. MT conducted the time-lapse *in vitro* imaging. SB, CB, and GS conducted and analyzed electrophysiological studies. ER and RM designed vectors and produced virus. ER conducted and analyzed gene expression studies. SB, MT, RM, and DP performed *in vivo* gene delivery studies. SB, MT, JY, EM, and DP performed histological processing and imaging. PK and DP performed quantitative stereological analysis. SB, GS, RM, and DP conceived and designed the study and contributed to the writing of the manuscript. All authors contributed to the article and approved the submitted version.

## Funding

This work was supported by NIH awards AG20047 and NS100514 to DP.

## Conflict of interest

The authors declare that the research was conducted in the absence of any commercial or financial relationships that could be construed as a potential conflict of interest.

## Publisher’s note

All claims expressed in this article are solely those of the authors and do not necessarily represent those of their affiliated organizations, or those of the publisher, the editors and the reviewers. Any product that may be evaluated in this article, or claim that may be made by its manufacturer, is not guaranteed or endorsed by the publisher.
