# Supplementary material for: Engineered neurogenesis in naïve adult rat cortex by Ngn2-mediated neuronal reprogramming of resident oligodendrocyte progenitor cells
Source: Front Neurosci. 2023 Aug 17;17:1237176. doi: 10.3389/fnins.2023.1237176 (PMC10471311; doi:10.3389/fnins.2023.1237176)
Supplement: Supplementary file 1 [file Data_Sheet_1.pdf]

Supplemental Video 1

Link: https://doi.org/10.5281/zenodo.8189544

Supplemental Video 1

Time-lapse imaging of the process of neuronal lineage re-specification of Ngn2-infected OPCs

Cultured OPCs were infected by retroviral delivery with Ngn2-GFP and imaged over 70 hours, beginning at 4 days post-infection until 7 days post-infection. Cultures were kept continuously for that period on a confocal microscope equipped with an environmental chamber to maintain normal incubator conditions. Imaging used both DIC optics for cell and process morphology and 488 nm laser excitation for detecting GFP-expression. There were also cells not infected with Ngn2 as indicated by the absence of GFP-expression. Three excerpts of the entire 70 hour recording are replayed at faster speed in the video to summarize activity at the beginning, the middle, and toward the end of the imaging period. In the first segment, cells that were GFP-positive and expressing Ngn2 began with a multipolar morphology and were active in moving about their environment. In the middle segment, many GFP-positive cells began to show morphological specification of their process and adopt a bipolar morphology. This morphology was quite fluid at this point as the processes were interacting with the environment and contacting other cells. By the final segment, GFP-positive cells showed more elaborate branching and continued to contact other cells while some processes and contacts appear to have stabilized. Upon removal from the microscope, cells were fixed and stained with antibodies to NeuN to detect a mature neuronal phenotype. Two fields of view of the immunostained cultures are shown with many GFP-positive cells colabled with NeuN (red) producing yellow cell bodies.
