# Supplementary material for: Engineered neurogenesis in naïve adult rat cortex by Ngn2-mediated neuronal reprogramming of resident oligodendrocyte progenitor cells
Source: Front Neurosci. 2023 Aug 17;17:1237176. doi: 10.3389/fnins.2023.1237176 (PMC10471311; doi:10.3389/fnins.2023.1237176)
Supplement: Supplementary file 2 [file Table_1.pdf]

Supplementary Table 1 Transcription Factors Used for In Vitro Screening

Transcription Factor Candidates		Source and Notes on Use
**Single Factors**		
	GFP Control	Addgene #16664
	Neurogenin2 (Ngn2)	B. Berninger; subcloned from p158
	NeuroD1	G. Chen
	Pax6	M.Götz and B. Berninger
	Dlx2	B. Berninger; Dlx2 vector (p59)
	Ascl1	B. Berninger; Ascl1 vector (p132)
**Co-Delivered Factors**		
	Ascl1 / Dlx2	As above; Combined 1:1
