# Supplementary material for: Engineered neurogenesis in naïve adult rat cortex by Ngn2-mediated neuronal reprogramming of resident oligodendrocyte progenitor cells
Source: Front Neurosci. 2023 Aug 17;17:1237176. doi: 10.3389/fnins.2023.1237176 (PMC10471311; doi:10.3389/fnins.2023.1237176)
Supplement: Supplementary file 3 [file Table_2.pdf]

Supplementary Table 2 List of Antibodies Used

Antibody	Species	Catalog #	Dilution	Manufacturer	Location
CamKIIα	mouse	MAB 8699	1:1000	Millipore	Billerica, MA, USA
ChAT	goat	Ab 101755	1:500	Abcam	Cambridge, UK
Doublecortin	guinea pig	AB 2253	1:1000	Millipore	Billerica, MA, USA
ED1 (CD68)	mouse	MCA241R	1:1000	Serotec	Oxford, UK
GAD67	mouse	MAB5406	1:2000	Millipore	Billerica, MA, USA
GFAP	rabbit	Z 0334	1:2000	Dako	Carpinteria, CA, USA
GFP	chicken	GFP 1020	1:5000	Aves	Tigard, OR, USA
Iba1	rabbit	019-9741	1:4000	Wako	Richmond, VA, USA
MAP2	rabbit	AB 5622	1:1000	Millipore	Billerica, MA, USA
Nestin	mouse	MAB 353	1:500	Millipore	Billerica, MA, USA
NeuN	mouse	MAB 377	1:2000	Millipore	Billerica, MA, USA
NeuN	rabbit	ABN78	1:2000	Millipore	Billerica, MA, USA
NG2	rabbit	AB 5320	1:200	Millipore	Billerica, MA, USA
O4 (IgM)	mouse	MAB 1326	1:400	R & D Systems	Minneapolis, MN, USA
Olig2	rabbit	AB9610	1:1000	Millipore	Billerica, MA, USA
PDGFRα	rabbit	PA5-17623	1:200	Pierce	Rockford, IL USA
PDGFRα	rabbit	Ab 32570	1:500	Abcam	Cambridge, UK
RFP	rabbit	Ab 62341	1:500	Abcam	Cambridge, UK
RIP	mouse	MAB 1580	1:500	Milllipore	Billerica, MA, USA
S100β	rabbit	04-1054	1:5000	Millipore	Billerica, MA, USA
Sox10	guinea pig		1:1000	M. Wegner, Gift	Erlangen, Germany
Synaptophysin	mouse	MAB 638	1:1000	Millipore	Billerica, MA, USA
Tbr1	rabbit	Ab 31940	1:500	Abcam	Cambridge, UK
vAChT	guinea pig	AB 1588	1:1000	Millipore	Billerica, MA, USA
vGLUT1	guinea pig	AB 5905	1:1000	Millipore	Billerica, MA, USA
β-III-tubulin	mouse	G7121	1:1000	Promega	Madison, WI USA
